# Web-based volume slicer for 3D electron-microscopy data from EMDB

**DOI:** 10.1016/j.jsb.2016.02.012

**Published:** 2016-05

**Authors:** José Salavert-Torres, Andrii Iudin, Ingvar Lagerstedt, Eduardo Sanz-García, Gerard J. Kleywegt, Ardan Patwardhan

**Affiliations:** a Protein Data Bank in Europe, European Molecular Biology Laboratory, European Bioinformatics Institute, Wellcome Genome Campus, Hinxton CB10 1SD, United Kingdom; b Present address: Universitat Politècnica de València, DISCA, Edificio 1G – Lab 3S-15, Camino de Vera S/N, 46022 València, Spain

**Keywords:** CSS, Cascading Style Sheets, DOM, Document Object Model, EMDB, Electron Microscopy Data Bank, EBI, European Bioinformatics Institute, NMR, Nuclear Magnetic Resonance, OME, Open Microscopy Environment, OMERO, OME Remote Objects, PDB, Protein Data Bank, PDBe, Protein Data Bank in Europe, SVG, Scaled Vector Graphics, 3DEM, Three-Dimensional Electron Microscopy, Electron Microscopy, Visualisation, Web components

## Abstract

We describe the functionality and design of the Volume slicer – a web-based slice viewer for EMDB entries. This tool uniquely provides the facility to view slices from 3D EM reconstructions along the three orthogonal axes and to rapidly switch between them and navigate through the volume. We have employed multiple rounds of user-experience testing with members of the EM community to ensure that the interface is easy and intuitive to use and the information provided is relevant. The impetus to develop the Volume slicer has been calls from the EM community to provide web-based interactive visualisation of 2D slice data. This would be useful for quick initial checks of the quality of a reconstruction. Again in response to calls from the community, we plan to further develop the Volume slicer into a fully-fledged Volume browser that provides integrated visualisation of EMDB and PDB entries from the molecular to the cellular scale.

## 1 Introduction

Over the past decade cryo-electron microscopy and electron tomography have become increasingly important tools in the arsenal of structural biology. Traditionally, these techniques complemented the more established approaches of X-ray crystallography and Nuclear Magnetic Resonance (NMR) spectroscopy by allowing larger structures, pleomorphic structures and structures in the cellular context to be studied albeit at poorer resolutions but without the need for crystals or high concentrations. Recent technological advances such as the introduction of direct electron detectors have revolutionised the field and led to the *de novo* determination of large complexes at near-atomic resolution, further underpinning the importance of these techniques for structural biology (Bai et al., 2015).

The Electron Microscopy Data Bank (EMDB) archive (Tagari et al., 2002) was established at the European Bioinformatics Institute (EBI) in 2002 and is the authoritative source for 3DEM data. Against the backdrop of technological advances, EMDB has experienced rapid growth and now contains over 3400 structures derived from a range of 3DEM techniques including single-particle reconstruction, helical reconstruction, tomography and electron crystallography (http://pdbe.org/emdb). The data archived in EMDB finds many uses. 3D reconstructions can be viewed in conjunction with published results to further analyse or corroborate claims made by the authors. They may be used to boot-strap single-particle image processing, compared with similar structures to examine structural changes, and fitted into reconstructions of larger structures such as cellular tomograms to further aid in their interpretation. They can be used for teaching and training purposes and by methods developers to refine, test or implement new algorithms.

The Protein Data Bank in Europe (PDBe; (Velankar et al., 2016)) currently provides a wide range of web-based services for searching (EMSearch – http://pdbe.org/emsearch, EMStats – http://pdbe.org/emstats; (Gutmanas et al., 2014)), and validating and visualising EMDB data (visual analysis pages, volume viewer, slice viewer; (Gutmanas et al., 2014, Lagerstedt et al., 2013)). Key to PDBe’s mission of “*Bringing structure to biology*” is the development of web-based resources that make it easier for expert and non-expert users alike to access and exploit structural data in EMDB, and to integrate it with data from other structural archives, such as the PDB, and with other bioinformatics resources (Gutmanas et al., 2013). PDBe engages regularly with relevant user communities to understand their needs and requirements with regards to archiving and resources for structural data. Notably, PDBe has organised several expert workshops in recent years to focus on specific issues including “*Data-Management Challenges in 3D Electron Microscopy*” (Patwardhan et al., 2012) and “*A 3D Cellular Context for the Macromolecular World*” (Patwardhan et al., 2014). These community interactions have identified a need for improved interactive visualisation of 2D slice images from 3D maps. Viewing individual slices provides a very simple means for users to assess the quality of maps and details of the processing such as the use of masking. Hitherto the EMDB slice viewer was the only web-based tool available for this purpose but it was restricted in that it was only available for tomograms in EMDB (which represent a minority of the entries) and only allowed viewing of slices in one direction (Lagerstedt et al., 2013). We have now developed a “Volume slicer” web service, which is available for all EMDB entries and allows for interactive visualisation of 2D slices in three orthogonal directions. In this paper we describe the design and implementation of, and future prospects for the Volume slicer.

## 2 User’s perspective

The Volume slicer is available for all EMDB entries from URLs of the form http://pdbe.org/EMD-####/3dslice (where EMD-#### is an EMDB accession code, e.g., EMD-2363), Fig. 1a. The Volume slicer can be used to examine the definition of structures in detail providing, for instance, more information on artefacts and resolution variation within a structure, Fig. 1b. The Volume slicer consists of a main viewing panel showing a 2D slice from the 3D map, a 3D navigation cube and thumbnail images for orientation and navigation, *min* and *max* density range sliders with a density histogram, and summary information about the entry. The image in the main viewing panel can be zoomed by using the slider to the right of the image; left clicking on a point will re-centre the view on that point if possible given the chosen zoom level. The 3D cube shows the location of the slice being examined in the main viewing panel as a rectangle. The three sliders or input boxes can be used to move the rectangle and will update the slice shown in the main viewing panel. Selecting one of the three radio buttons in the thumbnail panel changes the active viewing direction. The area shown in the main viewing panel is highlighted in orange in the thumbnail panels; the area can be changed with a left-mouse click on the thumbnail images.

## 3 Design overview

Web-based visualisation resources for EMDB provide easy, quick and convenient views of the data without requiring the EM volume to be downloaded (Lagerstedt et al., 2013). A common use case is when a user wants to inspect EMDB entries cited in a publication. For more detailed analysis users will typically employ advanced visualisation programs like Chimera (http://www.cgl.ucsf.edu/chimera; (Pettersen et al., 2004)) that use downloaded volumes. Although web-based resources do not explicitly involve data download, data still needs to be transferred from server to client. Given that typical EMDB datasets range in size from 100 MB to several GB, transferring entire datasets would be slow for most users with typical network connections. Furthermore, most web browsers impose varying memory restrictions often not exceeding 128 MB, and at present web-based front-end technologies are simply incapable of handling these large quantities of data efficiently. Such problems can be overcome by use of a technology that can extract the required 2D view from the 3D dataset on the server side and only transfer this to the client. A potential downside is inferior responsiveness – requests for changing views have to be communicated back to the server, and 3D transformations can be computationally expensive even on the server side. A balance therefore needs to be struck between the range of features offered to the user in terms of dynamically altering the views on the 3D data and the interactivity of the web-service. This balance is highly dependent on the available technological solutions and IT infrastructure.

PDBe has previously worked with the Open Microscopy Environment (OME) team to develop a slice viewer for tomograms (Lagerstedt et al., 2013). This slice viewer shows 2D sections from a 3D dataset, with functionality to interactively scroll through the slices. We used the OMERO back-end technology, which implements a client-server system where only 2D images are sent to the client (Allan et al., 2012). OMERO was originally developed for light and fluorescence microscopy data, but has gradually been adapted and extended to work with many forms of data (including EM data) through the BioFormats library (Linkert et al., 2010). Since the introduction of the slice viewer we have received user feedback encouraging development along the following lines: (a) making the viewer available for all EMDB entries, not just the tomograms, (b) making the interface more user-friendly, and (c) providing the functionality to view slices in more than one direction, preferably with arbitrary rotation. Of these requests the last one is, as expected, the most challenging. We searched for other web-based solutions that might be useful in this context but were unable to find any that could work efficiently in a multi-user environment and on a set of over 3000 highly varied EM structures. One particular solution that was considered was the Mouse Atlas from the Baldock group (http://www.emouseatlas.org/eAtlasViewer_ema/application/ema/anatomy/EMA49_3D.php; (Richardson et al., 2014)). This viewer allows for arbitrary rotation and slicing of a small set of mouse models by multiple users. However, this functionality needs to be assessed in light of the EMDB use case where any of over 3000 EM structures could be viewed simultaneously by multiple users. Providing views with arbitrary rotation and translations would involve intensive runtime computations to interpolate a slice from a 3D volume. This functionality was deemed unrealistic at the present time given the infrastructure available. Instead, we chose a less demanding solution by providing views along three orthogonal directions. Even this could be challenging if the rotations had to be performed at runtime. We therefore decided to use OMERO for the back-end, to pre-calculate and prepare the two additional orientations for each EMDB entry and to load them into OMERO. We designed the interface so that it is easy for the users to orient themselves in 3D space and to switch between the viewing directions, Fig. 1a.

Our method of user-based design involved creating a prototype, testing it on a small group of prospective users from the EM community, making changes based on their feedback and then iterating the process. Users involved in the testing included groups at MRC Laboratory of Molecular Biology (Cambridge), Birkbeck University of London and The Francis Crick Institute (London), and the backgrounds of these users spanned the full gamut from molecular to cellular EM. The testing protocol involved asking the user to perform certain tasks (for example, examining a certain feature in a structure) and monitoring (with as little help as possible) how they went about performing the task. We were encouraged by the fact that after a few iterations new users were having much fewer problems using the Volume slicer in general and in particular that issues that had been identified in early testing iterations no longer caused problems.

## 4 Preparation of data

EMDB map files need to be processed before they are suitable for display in the Volume slicer. All the steps involved in this processing are done with different programs of the IMOD 4.8 suite of tools (http://bio3d.colorado.edu/imod/; (Kremer et al., 1996)). Our choice of this package was motivated by the fact that it performed robustly over a wide range of maps, even those that were several GB in size.

## 5 Preparation procedure

(1) The (*fast*, *medium*, *slow*) axis order is checked in the header of the EMDB map file. The volume data codification is reordered if necessary to match the (*X*, *Y*, *Z*) axis ordering. Different combinations of the **clip** (**flipxy**, **flipyz**, **flipzx**) command are used depending on the original axes order. This IMOD command flips the image around various axes, obtaining the desired effect.
(2) The volume is rotated to match the three points of view that will be available in the Volume slicer (the top, front and right views). This is done using the **rotatevol** command with different angles, generating three volume files. A correction is performed in these rotations to set the original *Z*-axis pointing towards the top view, which is the common stack representation of EM images.
(3) To create thumbnail volumes for the three different views, the rotated volumes are scaled with the **squeezevol** command.
(4) The three rotated volumes and the corresponding thumbnail volumes for each EMDB entry are imported into the OMERO server data repository, to make them available to the Volume slicer client application.

## 6 OMERO image server

The Volume slicer uses the OMERO server to store and provide slice data from the 3D volumes to the client. The server renders a compressed *jpeg* image that is sent to the client application. The OMERO server calculates the *jpeg* images for every web query, but implements a cache to minimise the need for repeated calculation of the same images. The *jpeg* compressed images range from a few kB to a couple of MB in size, depending on the size of the original volume and the zoomed region being displayed. For example, entry EMD-1273 is a large tomogram 2048 × 2048 × 76 voxels in size. When fully zoomed out, the size of a compressed *jpeg* of a whole slice is 2.8 MB (highest *jpeg* quality setting).

Sending such large images to the client can be inefficient in that the client web browser in any case has to resample these large images to fit the size of the visualisation area. One solution would be to down-sample the image on the server side to match the size of the display area, but this would increase the server response time. A more efficient approach that we exploit is to selectively change the compression quality with the zoom level – the quality of the *jpeg* is progressively increased as the user zooms into a region and results in the transfer of approximately the same amount of data regardless of the zoom. As an example, the size of images sent to the client for EMD-1273 is always around 300 kB.

## 7 Web application

**Fig. 2:**
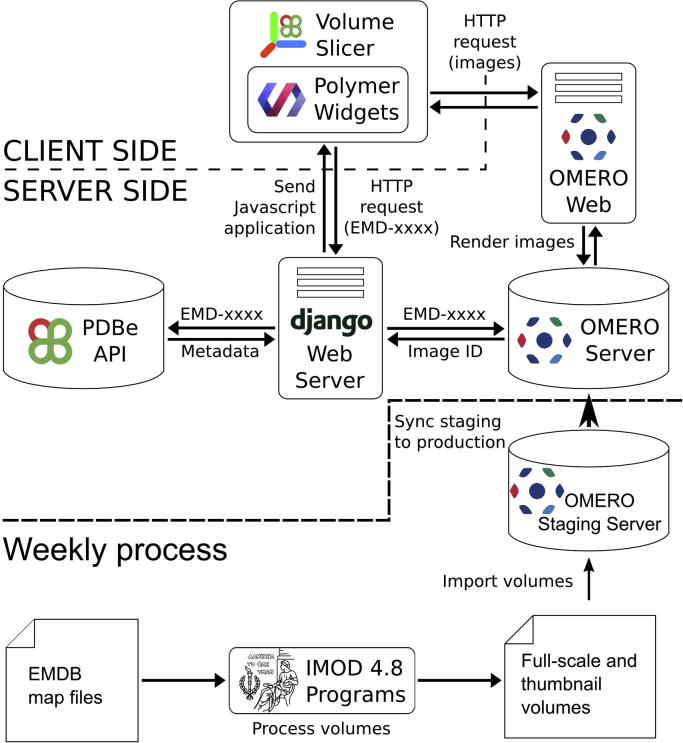
Overview of the processes and infrastructure at PDBe that underpin the Volume slicer. Once a week new and modified entries in EMDB are checked, and the volumes are prepared using IMOD and loaded into the OMERO staging server, which is then synchronised to the public servers at the time of the weekly EMDB release. The application is implemented using Polymer web components in the front-end and the OMERO.web application and the Python/Django application in the back-end. The OMERO.web application acts as an API handling requests from the client and serving data from the OMERO server.

The web application back-end has been implemented as a Python/Django application (https://www.djangoproject.com/) in the context of a wider Python/Django project that serves as the code-base for most of the EM resources provided by PDBe, Fig. 2. The actual Volume slicer page has been implemented using HTML 5 and the Polymer 1.0 library (https://www.polymer-project.org/1.0/). Polymer is built on top of web components standards and provides the functionality to define custom HTML elements. The Javascript polyfills API (http://webcomponents.org/polyfills/) enables programs using web components to run in browsers that lack support for native web components standards.

The Polymer library allows developers to implement reusable page components, or widgets, as they are commonly known, as custom HTML elements. The “shadow DOM” enables the containment of Cascading Style Sheets (CSS; http://www.w3.org/Style/CSS/Overview.en.html) definitions to the scope of the widget thus preventing conflicts with other page elements. The double data binding and the observer interface enable communication between widgets. When modified, parameters such as the slice position, zoom and density range are propagated automatically across all the widgets in the page. Furthermore, we found the data-binding syntax very convenient for modifying the Scaled Vector Graphics (SVG; http://www.w3.org/Graphics/SVG/) images dynamically.

We have developed five custom elements for the Volume slicer page (Fig. 1a). The **information panel** displays basic information about the entry that is obtained from the EMDB API (http://www.ebi.ac.uk/pdbe/api/doc/emdb.html). The **navigation panel** has sliders and input boxes to control the axis positions and a three-dimensional SVG representation of the current slice and zoomed area. There are three view panel widgets, one for each of the orthogonal orientations. The **view panels** display thumbnail images with SVG overlays marking the zoomed area. The **main visualisation panel** shows the active slice region and has a slider to change the zoom level of the image shown. The **density range panel** shows the map-density histogram and two sliders to set the minimum and maximum density range. The histogram information is obtained from the EMDB API. The mean of the distribution is represented in the graph with a green line. The *y*-axis is on a logarithmic scale. The navigation panel, view panels, main view panel and density range panel are all coupled and respond to user events, for example, moving a slider in the navigation panel will update the image in the view panels and the main navigation panel. Images for the main panel and the thumbnails are fetched from the OMERO server.

## 8 Map-density distribution

Voxels in an EMDB map file can be of any of the types allowed in the CCP4 format specification (http://www.ccp4.ac.uk/html/maplib.html) from bytes to 32-bit floating point values. The OMERO server converts these to the 8-bit range of the *jpeg* format used before they are sent to the client. Masking, noise, background variations and fiducial markers often determine the extreme map-density values in an EM map. Mapping the full density range onto an 8-bit representation can often lead to loss of meaningful density variations relating to the structure. The density-range panel with its histogram and sliders makes it possible to adjust the range to highlight relevant features.

For the Volume slicer we have addressed the issue of the default *min* and *max* densities for the linear grey-level mapping. If we were to simply take the full dynamic range then there is a risk that many inexperienced users, especially those from other domains of biology, will be confused by the apparent lack of meaningful features.

We have studied the map-density distributions of maps in EMDB, keeping in mind that reconstructions from different methods, e.g. single particle and tomography, may exhibit different characteristics. A common characteristic of most maps, with the exception of tomograms, is a strong peak in the map-density distribution, often at zero density. This peak is due to the masking that has been applied to the map and has the effect of shifting the mean value and reducing the variance of the map-density distribution. If we use a variance-based measure to calculate the default *min* and *max* density values for the initial view, these effects make it very difficult to come up with a simple scheme that can work for a broad range of maps. Taking the *min* and *max* as ±2 *σ* (standard deviations) from the mean density works well for tomograms, but causes saturation to varying extents on many single particle maps. We therefore exclude the masking peak (the most populated density bin, ±2 bins) from the density distribution prior to further statistical analysis. In one attempt we tried to use the mean ± various multiples of the standard deviation. Again this worked reasonably well for tomograms but not for single-particle reconstructions where there was very often a noticeable skewness increasing the distance between the mean and the mode. If we used the mode instead of the mean there was an improvement in the visualisation of many single-particle maps, but there were cases where the distribution was seemingly bi-modal and using the mode would have yielded worse results. Finally, after many tests we found that if we looked at the cumulative density distribution and set the default *min* and *max* at the 0.5th and 99.5th percentiles of the number of voxels (saturating only 1% of the voxels) then this worked well across a broad range of maps, stretching the contrast but with little saturation.

## 9 Production process

EMDB is updated weekly with new entries being released every Wednesday at 00:00 UTC (Lawson et al., 2011). Following the update, we synchronise externally visible OMERO production servers with internal staging servers with the new and updated data. The internal staging server is loaded with new data the preceding Sunday, allowing for two days to check and reload data in case of errors or failures. The loading process includes the volume preparations described in “Preparation of data”. As many EMDB maps are quite large, these preparations are done by submitting jobs to the EBI clusters and managing the jobs using an in-memory redis (http://redis.io/) data store. The process is robust in the sense that failed jobs will be tracked and retried a number of times.

## 10 Future prospects

The Volume slicer is now available from the PDBe website for nearly all EMDB entries. It uniquely allows users to view slices from 3DEM reconstructions in three orthogonal directions in a web browser, without the need to download any desktop application or a whole EM volume. We integrated user-experience testing in our development process to ensure that we were building a user-centric and relevant resource. For example, early on we received feedback that the navigation panel was too cluttered (it showed a cube for the whole volume which made it difficult to discern the active plane). As a consequence we removed the cube and simplified the navigation panel. Similarly there was a question of whether it was better to have the thumbnail views to the left (as it is now) or in the bottom left corner and the outcome from A/B testing was a small preference for having them on the left.

As mentioned earlier, it is important that the default view shown when the Volume slicer is initially loaded is meaningful. This was the motivation for our efforts to derive sensible *min* and *max* values for the density mapping. Similar considerations could be made in terms of the slice shown in the main panel by default – currently it is the central slice in the ‘*z*’ direction. We are considering using the density variance in each slice to select by default the most “informative” slice, and possibly also to display the variance as a chart versus the slice number to enable rapid selection of “interesting” slices.

Additional features suggested by members of the EM community include a facility to reverse the contrast of an image (currently in testing) and to provide non-planar sections (cylindrical or spherical) from the reconstruction. We will normally prioritise the implementation of features suggested by several users and corroborated by user testing. We therefore encourage users to contact us regarding potential use cases and improvements that could be made.

We are aware that the first port of call for information on EMDB entries for many users will not necessarily be the PDBe website but other resources such as journal publications. While in-bound links are one way for users to navigate to the Volume slicer, the design of the page in terms of Polymer web components lends itself to easy integration of the Volume slicer widgets into other web resources.

Our vision for the future is to transform the Volume slicer into a “Volume browser” that will integrate views of structural data on different scales and from both the EMDB and PDB archives (Gutmanas et al., 2013). There are major challenges that must be overcome to achieve this. To accommodate coordinate model visualisation we will need to integrate an interactive 3D viewer that can also show density maps. We have previously developed a Java-based viewer that does this (Lagerstedt et al., 2013) but given the diminishing support for Java applets in modern browsers it is becoming increasingly likely that we will need to consider other solutions such as WebGL (e.g., iview (Li et al., 2014); http://istar.cse.cuhk.edu.hk/iview/). Integrated visualisation of cellular and molecular structure data will require overlaying segmentations onto map slices in the Volume browser. Unfortunately, support for segmentations in EMDB is at best rudimentary and they are not adequately annotated in terms of links to other structural and bioinformatics resources. This deficiency was identified and discussed at two expert workshops – “*Data management challenges in 3D Electron Microscopy*” (Patwardhan et al., 2012) and “*A 3D cellular context for the macromolecular world*” (Patwardhan et al., 2014) – where PDBe was strongly encouraged to take concrete steps to improving the support for segmentations in EMDB and we have now obtained funding to do so. We will continue to engage with the EM community to ensure that the Volume slicer/Volume browser serves their needs and becomes a valuable resource for the visualisation and analysis of structural data on a range of scales.

## Competing financial interests

We have read and understood JSB policy on declaration of interests and declare that we have no competing interests.

## Figures and Tables

**Fig. 1a:**
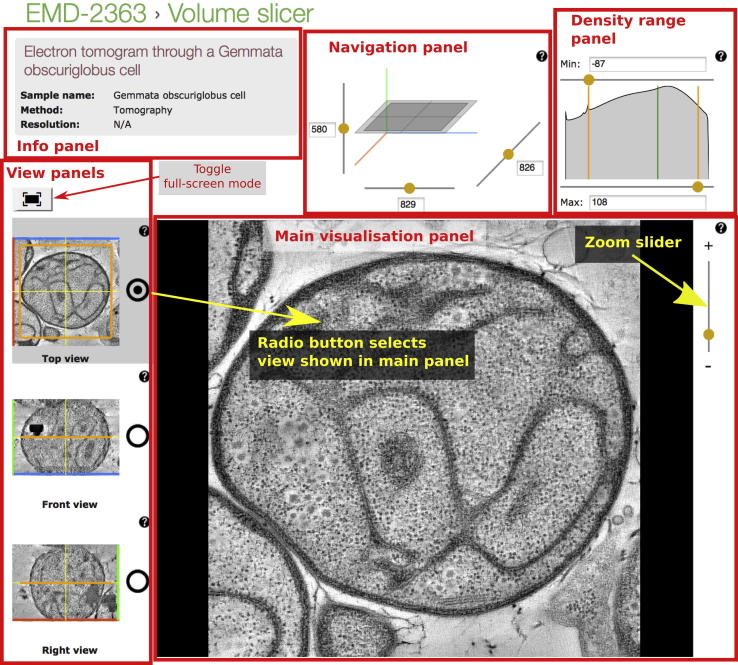
The Volume slicer page for EMD-2363 (pdbe.org/emd-2363/3dslice; (Santarella-Mellwig et al., 2013)). The navigation panel and view panels can be used to change the slice shown in the main visualisation panel. The three view panels show orthogonal slices from the 3D volume centred at the origin of the plane shown in the navigation panel. The radio buttons can be used to change the active view orientation shown in the main panel. Full-screen mode can be toggled using the button above the view panels. The density-range panel shows the density histogram for the volume and the *min* and *max* sliders allow the mapping to the display grey scale range to be adjusted.

**Fig. 1b:**
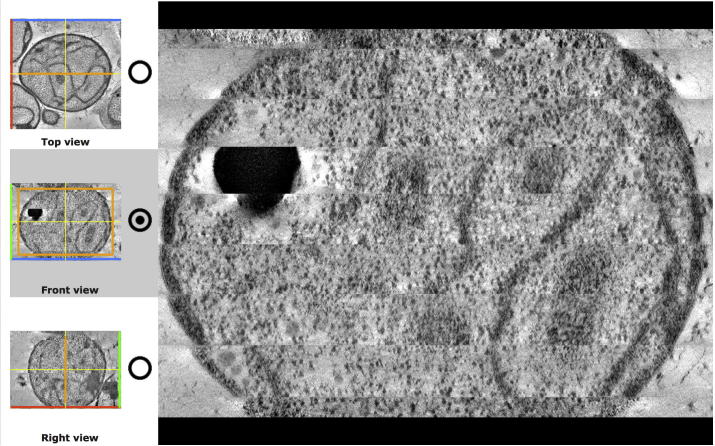
Example application of the Volume slicer. The same entry as in Fig. 1a is viewed using the “Front view”. The artefacts created when stitching together serial section tomography reconstruction slabs become clearly visible in the Volume slicer, when the reconstruction is viewed from a direction orthogonal to the axis of sectioning.
